# Insights into the Potential of Biopolymeric Aerogels as an Advanced Soil-Fertilizer Delivery Systems

**DOI:** 10.3390/gels9080666

**Published:** 2023-08-17

**Authors:** H. P. S. Abdul Khalil, Kanchan Jha, Esam Bashir Yahya, Sandeep Panchal, Nidhi Patel, Arindam Garai, Soni Kumari, Mohammed Jameel

**Affiliations:** 1 Bioresource Technology Division, School of Industrial Technology, Universiti Sains Malaysia, Gelugor 11800, Penang, Malaysia; kanakjha.jha20@gmail.com (K.J.); nidhi.patel1000.np@gmail.com (N.P.); sonithakur@student.usm.my (S.K.); 2 Green Biopolymer, Coatings and Packaging Cluster, School of Industrial Technology, Universiti Sains Malaysia, Gelugor 11800, Penang, Malaysia; 3 Bioprocess Technology Division, School of Industrial Technology, Universiti Sains Malaysia, Gelugor 11800, Penang, Malaysia; 4 Department of Civil Engineering, Government Polytechnic Mankeda, Agra 283102, Uttar Pradesh, India; 2290sandy@gmail.com; 5 Department of Mathematics, Sonarpur Mahavidyalaya, Kolkata 700149, West Bengal, India; fuzzy_arindam@yahoo.com; 6 Department of Civil Engineering, College of Engineering, King Khalid University, Abha 61421, Asir, Saudi Arabia; jamoali@kku.edu.sa

**Keywords:** soil fertilizers, biopolymers, aerogels, fertilizer delivery, encapsulation, slow release

## Abstract

Soil fertilizers have the potential to significantly increase crop yields and improve plant health by providing essential nutrients to the soil. The use of fertilizers can also help to improve soil structure and fertility, leading to more resilient and sustainable agricultural systems. However, overuse or improper use of fertilizers can lead to soil degradation, which can reduce soil fertility, decrease crop yields, and damage ecosystems. Thus, several attempts have been made to overcome the issues related to the drawbacks of fertilizers, including the development of an advanced fertilizer delivery system. Biopolymer aerogels show promise as an innovative solution to improve the efficiency and effectiveness of soil-fertilizer delivery systems. Further research and development in this area could lead to the widespread adoption of biopolymer aerogels in agriculture, promoting sustainable farming practices and helping to address global food-security challenges. This review discusses for the first time the potential of biopolymer-based aerogels in soil-fertilizer delivery, going through the types of soil fertilizer and the advert health and environmental effects of overuse or misuse of soil fertilizers. Different types of biopolymer-based aerogels were discussed in terms of their potential in fertilizer delivery and, finally, the review addresses the challenges and future directions of biopolymer aerogels in soil-fertilizer delivery.

## 1. Introduction

Fertilizers are substances added to soil or crops to enhance plant growth and increase crop yields [1]. They are commonly used substances that are applied to soil or crops with the aim of promoting plant growth and improving crop yields. Their primary function is to supply essential nutrients such as nitrogen, phosphorus, and potassium, which are vital for the overall health and wellbeing of plants, facilitating their optimal development and productivity [2,3,4,5]. The use of fertilizers has played a critical role in increasing agricultural productivity and meeting the food demands of a growing population [1]. By incorporating various types of soil fertilizers, numerous researchers have made significant improvements in enhancing the productivity of diverse plants [6,7,8]. Thus, fertilizers have become increasingly important as the global population continues to grow, placing greater pressure on agriculture to produce more food. The United Nations predicts that the global population will reach 9.7 billion by 2050, which means that food production will need to increase by 70% to meet the demand [9]. It has been reported that fertilizers can help achieve this goal by improving crop yields and making agriculture more efficient [10,11]. The demand for fertilizers has increased as a result of the growing global population and the need to produce more food. In addition, the demand for biofuels has also increased the demand for fertilizers as crops, such as corn and soybeans, are used to produce biofuels [12]. However, the excessive use and misuse of soil fertilizer also raise concerns about environmental impacts, including soil and water pollution, greenhouse gas emissions, and other negative environmental effects [13,14,15]. Despite the environmental impact, there has been an expansion in the production of synthetic fertilizers, which are manufactured using chemical processes to meet the increased demand for fertilizers [15].

Several technologies and tools have been proposed and used to limit the adverse environmental impact of fertilizers and implement controlled fertilizer delivery, including soil sensors [16], weather stations [17], and computer software that can analyze data and adjust the amount and timing of fertilizer delivery based on real-time conditions [18]. Controlled fertilizer delivery refers to a system or method that regulates the amount of fertilizer delivered to crops over a specific period. The goal of this approach is to provide the crops with the appropriate amount of nutrients they need at each stage of growth or for a constant period of time [19]. Controlling the release rate of fertilizers can effectively offer multiple environmental, economic, and yield benefits [20].

The conventional fertilizer coating involves spraying molten elemental sulfur over urea granules (the fertilizer), followed by a sealant wax coating to close any cracks, which are associated with several issues, including the inability to control release, since wax is not soluble in water, in addition to the issue of the excessive use of sulfur [21]. A few years later, a thin layer of organic polymers was used to cover the sulfur instead of the sealant wax [22]. Other researchers have used inorganic polymers, which are resin-based on the surface of the fertilizer granule, and reported a slower release rate [23]. The material used for coating should not be toxic to the plants and the environment, as it will sustain the fertilizer release and remain in the soil for the long term. In recent years, biopolymer aerogels have been proposed as novel delivery systems to provide prolonged and controlled release of pharmaceuticals and other bioactive compounds [24]. Several review articles have been published about the properties of biopolymer aerogels [25,26], their modifications [27,28], and different applications [29,30,31]. Specific application of these materials, such as their use in drug delivery applications, has been also extensively reviewed, including nanocellulose-based aerogels [32], chitosan-based aerogels [33], gelatine-based aerogels [34], starch-based aerogels [35], alginates-based aerogels [36], and general biopolymer aerogels [37]. However, no single review paper has been published to discuss the role or potential of biopolymer-based aerogels in soil-fertilizer delivery. In the present review, we highlight types of soil fertilizers, their properties, and adverse health and environmental effects. The review also discusses the properties and potential of biopolymeric aerogels in soil-fertilizer delivery and addresses the future directions and challenges that face biopolymer aerogels in soil-fertilizer delivery applications.

## 2. Properties of Biopolymeric Aerogels

**Figure 1:**
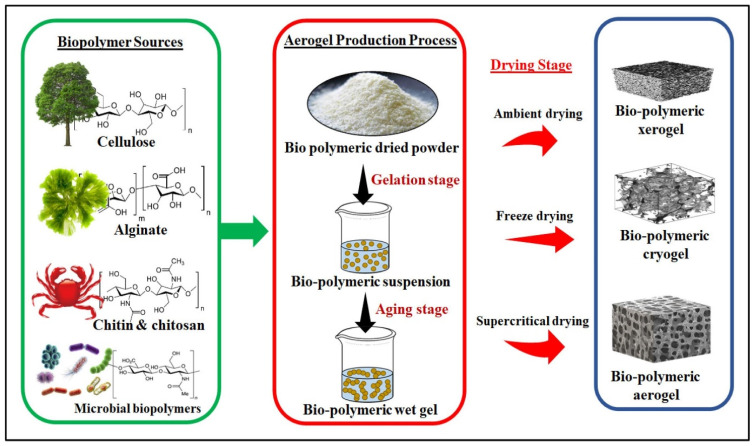
Schematic drawing of the main principle of biopolymeric aerogel fabrication. Reprinted with permission from Ref. [37]. 2023, Elsevier.

Biopolymeric aerogels are a type of aerogel that is made from biopolymers, which are natural polymers derived from living organisms such as plants (such as cellulose, alginates, starch, etc.), animals (such as chitosan, collagen, gelatine, etc.), and bacteria (such as bacterial cellulose and polyamides) [37]. Biopolymeric aerogels have several advantages over traditional aerogels, including being more environmentally friendly, biodegradable, and renewable [38]. The process for making biopolymeric aerogels involves creating a biopolymeric hydrogel using a biopolymer solution, then removing the liquid from the gel through a process called supercritical drying [39], or freeze-drying, as shown in Figure 1 [40].

This process results in a highly porous, lightweight material with excellent properties [29]. The properties of aerogel are influenced by various factors that play a crucial role in its formation and final characteristics. These factors include the choice of precursor materials, their concentration, and the mixing ratio used during gel formation [41,42]. The drying process employed also significantly impacts the resulting properties of aerogel [43]. Understanding and controlling these factors enable researchers to tailor the desired properties of aerogel, such as porosity, density, thermal conductivity, and mechanical strength [44]. There are several types of biopolymers that can be used to make aerogels, including cellulose, chitosan, carrageenan, alginate, and proteins like collagen and gelatin [45]. Each type of biopolymer has its own unique properties that can be leveraged to create aerogels with specific properties and applications. Biopolymer aerogels have several unique properties that make them an attractive material for various applications (Table 1). Some of the properties of biopolymer aerogels include their high extremely high porosity with a high surface-area-to-volume ratio, which makes them ideal for applications such as drug delivery applications and thermal and acoustic insulation [46,47]. In a recent study, Setyawan et al. [48] fabricated hydrophobic cellulose-based aerogels with a relatively high porosity of 97.5% by using biomass coir fibers and a freeze-drying technique. The authors reported an ultralow density (ρ =  0.047 g/cm^3^) and excellent elastic properties for their fabrication. Owing to their ultralow density, biopolymer aerogels are extremely lightweight, making them useful in applications where weight is a concern, such as in aerospace engineering or wearable technologies [49]. Biopolymer aerogels are made from natural materials and are biodegradable, which makes them an environmentally friendly alternative to synthetic aerogels [50]. The biodegradation time of biopolymer aerogels can vary depending on various factors, including the specific biopolymer used, the environmental conditions, and the presence of microbial activity [51]. Generally, biopolymer aerogels are designed to be biodegradable and can undergo degradation within a range of several months to a few years [51,52]. However, the exact biodegradation time can differ significantly based on the composition and structure of the aerogel, as well as the specific conditions in which it is exposed to degradation agents [53]. Extensive research is being conducted to optimize the biodegradation properties of biopolymer aerogels for different applications and environments. Biopolymer aerogels can be engineered to have varying degrees of mechanical strength, from brittle to flexible, depending on the intended application [54]. Some biopolymers used to make aerogels, such as collagen or chitosan, are biocompatible and can be used in biomedical applications such as tissue engineering, drug delivery, or wound healing [24,55]. The properties of biopolymer aerogels can be tuned by varying the type and concentration of biopolymer used, as well as the processing conditions such as drying temperature or pressure [56,57]. This makes them versatile materials that can be tailored to specific applications [57]. Refer to Table 1 for the illustration of different biopolymer properties and applications.

## 3. Soil Fertilizers: Properties and Issues

The fertilizer market is surging, with an overall revenue growth expectation of hundreds of billions of dollars within the next ten years. The market is expected to grow to US$271.6 billion by 2030, more than the US$201.1 billion valuations in 2021, growing at a CAGR of 3.4% every year [66]. The rise of the agriculture sector is being driven by the expanding population. By 2050, the world’s population will have surpassed nine billion people, according to the United Nations. Furthermore, the Food and Agriculture Organization predicts that, by 2050, more than 70% of the world’s population would be living in cities [67]. Due to the lack of arable land around the world, farmers are being compelled to utilize fertilizers to boost their agricultural output. As a result, the worldwide fertilizer market would rise throughout the forecast period due to the rising population. The rise of other industries, such as agriculture and horticulture, is also propelling the global fertilizer industry forward. Governments all around the world are making significant investments in the global fertilizer market’s development [68].

### 3.1. Types of Common Fertilizers

**Figure 2:**
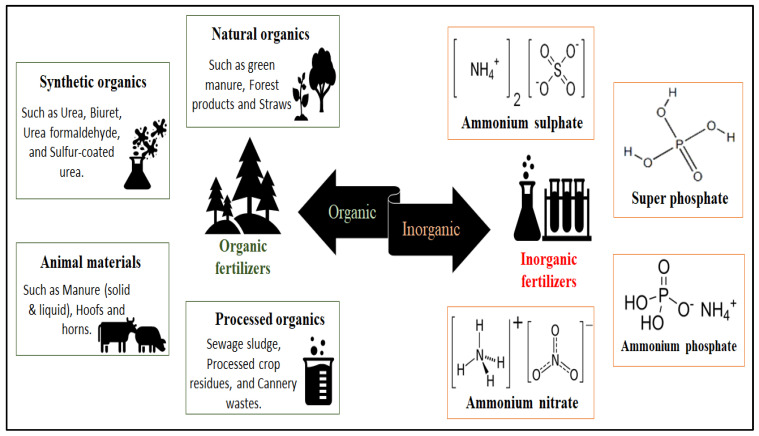
Illustration of different organic and inorganic fertilizers.

Fertilizers can be applied to soil or plants in various forms, including granular, liquid, and slow release [69]. Granular fertilizers are dry pellets that are applied directly to the soil, while liquid fertilizers are dissolved in water and applied directly to plants or soil [70]. In a recent study, Alameen and coworkers developed a control system for variable-rate application of a variety of granular fertilizers [71] that the authors were able to develop. As a variable-rate application is essential for optimum crop yield, the authors were able to adjust the manual fertilizer rate of the developed granular fertilizers with a very minor application-rate error. Liquid fertilizers are a type of fertilizer that comes in a liquid form and is used to provide essential nutrients to plants. These fertilizers are typically mixed with water and applied to the soil or directly to the plant leaves through a process known as foliar feeding [72]. Slow-release fertilizers are formulated to release nutrients over an extended period of time, reducing the need for frequent applications [73]. Generally, fertilizers can be classified as organic fertilizers and inorganic fertilizers, as presented in Figure 2.

Organic fertilizers are derived from natural sources, while inorganic fertilizers are those which are usually manufactured from synthetic sources and provide a quick release of nutrients [6]. It has been reported that organic fertilizers release nutrients slowly, improving soil structure and fertility over time [2,74]. They include natural organics, synthetic organics, animal materials, and processed organics. Natural organic fertilizers have been promoted to restore the health of the soil and enhance its fertility status [75]. Durán-Lara and coworkers stated that several organic materials can be used as natural fertilizers, including crop residues, animal manure, compost, vermicompost, and biochar [76]. Although these organic fertilizers are environmentally safe and nontoxic, they are limited and difficult to implement on a large-scale level. Processed organic fertilizers are fertilizers that are made from organic materials that have undergone some form of processing to improve their nutrient content or make them easier to handle and apply [77]. These fertilizers are derived from organic sources such as animal manure, plant residues, and food waste [78]. The processing methods may include composting, fermentation, vermicomposting, or bioconversion techniques [79]. Processed organic fertilizers offer several advantages, including improved nutrient release and uptake, reduced odor and weed seeds, and enhanced soil fertility and structure [80,81]. However, careful consideration must be given to the quality, nutrient balance, and application rates of processed organic fertilizers to ensure optimal results and minimize environmental impacts [78]. To enlarge the production and enhance the performance of fertilizers, several scientists have synthesized organic materials, such as urea, biuret, formaldehyde urea, and sulfur-coated urea, and used them as synthetic organic fertilizers.

On the other hand, inorganic fertilizers are fertilizers that are made from synthetic chemicals rather than organic materials. They are typically produced through industrial processes and contain specific amounts of nitrogen, phosphorus, and potassium, which are essential nutrients for plant growth [82]. Although, inorganic fertilizers have several advantages over organic fertilizers, including their precise nutrient content, long shelf life, and ease of application. They can also be more cost-effective than organic fertilizers in some cases [83]. One of the cost-effective issues associated with inorganic fertilizers is their initial purchase cost [84]. Inorganic fertilizers are often produced through industrial processes, requiring energy and resources, which contributes to their higher price compared to organic fertilizers [85,86,87]. Additionally, inorganic fertilizers may require more frequent applications due to their fast-release nature, leading to increased costs over time [88]. Furthermore, the transportation and distribution costs of inorganic fertilizers can add to their overall expense [84]. However, the integrated use of organic and inorganic fertilizers might solve some of these issues, which involves combining the benefits of both types of fertilizers to optimize nutrient management and improve crop productivity [89]. By blending organic and inorganic fertilizers, farmers can capitalize on the immediate nutrient availability of inorganic fertilizers while also harnessing the long-term soil-building properties of organic fertilizers [83,90]. This approach can enhance nutrient balance, promote soil health, and mitigate the potential negative impacts of excessive inorganic fertilizer use, such as nutrient runoff and environmental pollution [91]. Additionally, the integration of organic and inorganic fertilizers can offer a more sustainable and cost-effective solution by reducing the reliance on synthetic fertilizers and improving nutrient use efficiency in agricultural systems [92,93]. Proper nutrient-management practices and careful consideration of crop nutrient requirements are crucial for achieving successful integration and maximizing the benefits of both fertilizer types [92].

### 3.2. Environmental and Health Issues with Soil Fertilizers

**Figure 3:**
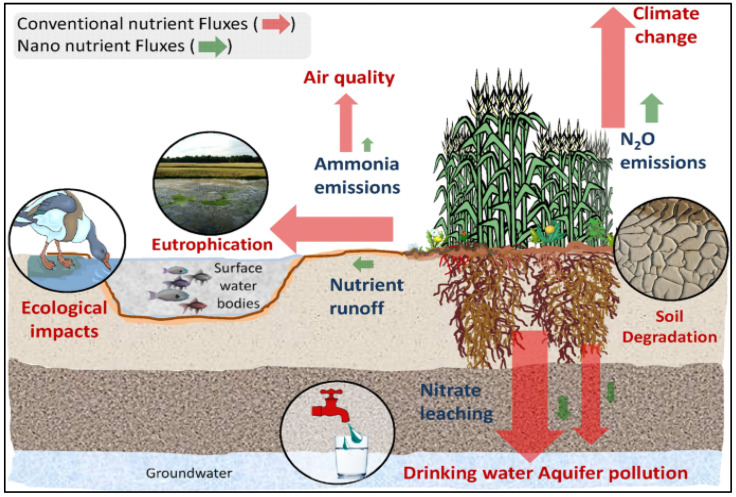
Illustration of fertilizers related to nutrient leakages and their environmental consequences. Reprinted with permission from Ref. [97]. 2022, Springer Nature.

The proper use of fertilizers can help to increase crop productivity, improve soil health, and protect the environment. Overuse or improper use of fertilizers can lead to soil degradation, which can reduce soil fertility, decrease crop yields, and degrade ecosystems [94]. Excess fertilizer can lead to soil acidification, which can reduce the availability of nutrients, increase soil erosion, and decrease water retention. Fertilizers can leach into waterways and cause eutrophication, which is an overgrowth of algae and other aquatic plants that can deplete oxygen levels and harm aquatic life [14,95,96]. Figure 3 illustrates the adverse environmental effect of soil fertilizers.

Nitrogen and phosphorus from fertilizers are the main contributors to eutrophication. The production, transportation, and application of fertilizers can result in air pollution from greenhouse gas emissions, including carbon dioxide and nitrous oxide. Nitrogen fertilizers can also contribute to air pollution by producing ammonia and nitrogen oxides, which can lead to acid rain and smog [88]. The production and use of fertilizers can contribute to climate change through the release of greenhouse gases, including carbon dioxide and nitrous oxide. The manufacture of nitrogen fertilizers is a particularly energy-intensive process that can lead to high carbon emissions [98,99]. The use of fertilizers can alter the nutrient balance in the soil and lead to changes in the composition of plant and animal communities. This can have negative effects on biodiversity and ecosystem services. To mitigate these environmental issues, it is important to use fertilizers judiciously and in a sustainable manner [14]. This can involve practices such as using organic fertilizers, reducing fertilizer use through precision agriculture techniques, and implementing nutrient-management plans to minimize nutrient runoff. It has been reported that fertilizer misuse can contribute to water pollution, primarily through a process called nutrient runoff [100]. When fertilizers are applied in excessive amounts or at inappropriate times, plants cannot absorb all the nutrients, such as nitrogen and phosphorus. These excess nutrients can easily wash away with rainfall or irrigation water. Through heavy rainfall or irrigation, the excess nutrients on the soil surface can be carried away by runoff water. This runoff can flow into nearby water bodies like rivers, lakes, and streams [101]. Fertilizer can also be transported through soil erosion. If soil is not properly managed, it can erode and carry away both the fertilizer and the topsoil-containing nutrients. When fertilizers are applied in soluble forms, such as nitrates and phosphates, they can dissolve in water and move downward through the soil. This process is called leaching. If the soil is porous or if excessive irrigation is applied, the nutrients can leach through the soil profile and reach groundwater sources.

## 4. Biopolymer Aerogels in Soil-Fertilizer Delivery

**Figure 4:**
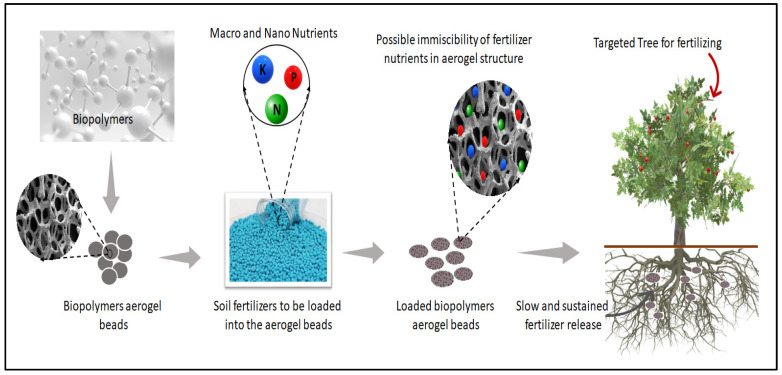
Schematic drawing of biopolymer-based aerogel beads in soil-fertilizer delivery.

Biopolymer aerogels have shown promising potential in various applications, including soil-fertilizer delivery. As the name suggests, they are aerogels made from biopolymers, which are natural polymers derived from renewable resources [102]. When it comes to soil-fertilizer delivery, biopolymer aerogels offer several advantages, including their ability to take, encapsulate, and coat fertilizers and/or nutrients and be used as carriers for controlled release [103]. The porous structure of aerogels allows for the encapsulation of fertilizers, protecting them from leaching or rapid degradation. The release of nutrients from the aerogels can be controlled by adjusting the composition and structure of the aerogel, allowing for a slow and steady release of fertilizers over an extended period, as shown in Figure 4 [25,37].

Biopolymer aerogels can also improve the efficiency of nutrient utilization in plants. By encapsulating fertilizers within the aerogel matrix, the release of nutrients can be synchronized with the plant’s needs. This reduces nutrient loss through leaching or runoff and minimizes the risk of overfertilization. The controlled release also ensures that the nutrients are available to the plants for a longer duration, maximizing their uptake and utilization [104]. Another advantage of biopolymer aerogels is their excellent water absorption and retention properties [28,105]. When incorporated into the soil, they can act as water reservoirs, absorbing and holding moisture. This helps to maintain optimal soil moisture levels, preventing water stress in plants and improving overall plant health. The water-retention capability of biopolymer aerogels can also reduce irrigation frequency and water consumption, making them environmentally friendly [30]. Several studies have reported the ability of biopolymer aerogels can enhance soil structure by promoting aggregation and reducing soil compaction [106,107]. When aerogels are added to soil, they can improve its porosity, allowing for better air circulation and root penetration. This leads to healthier root development and improved nutrient uptake by the plants. Additionally, the presence of aerogels can enhance the soil’s water-holding capacity, making it more resilient to drought conditions [108]. Biopolymer aerogels are typically derived from renewable resources such as cellulose, chitosan, or alginate. They are biodegradable and environmentally friendly alternatives to synthetic materials. When used as soil-fertilizer carriers, biopolymer aerogels eventually break down and become integrated into the soil, leaving no harmful residues [109].

### 4.1. Cellulose-Based Aerogel in Soil-Fertilizer Delivery

**Figure 5:**
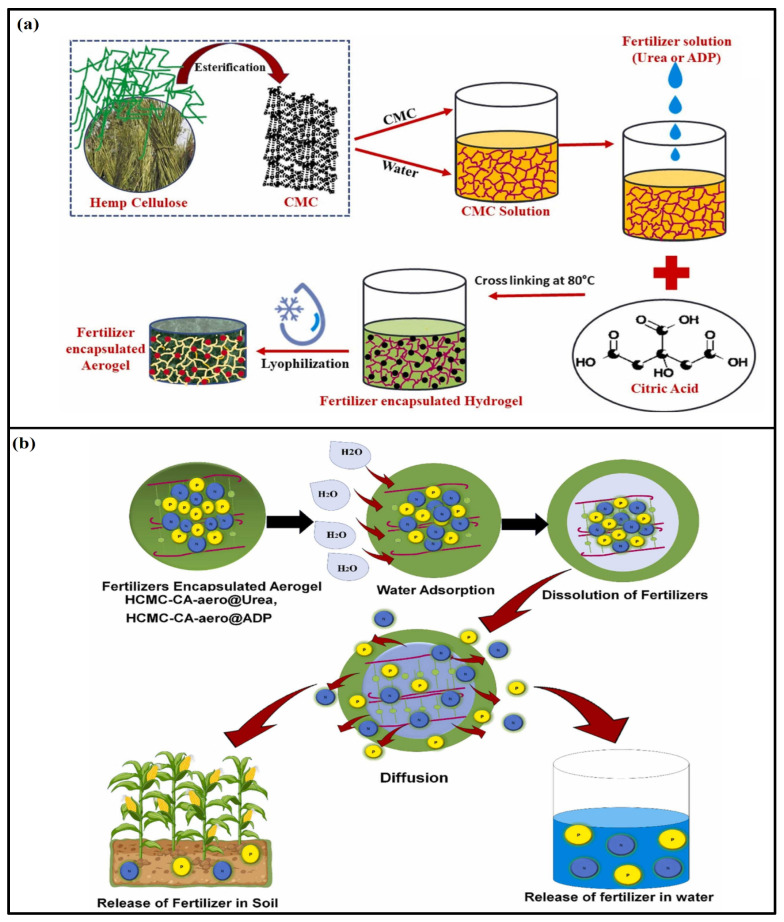
Cellulose-based aerogel in fertilizer delivery; (**a**) Extraction, conversion, and crosslinking of cellulose with citric acid and the fabrication of fertilizer-encapsulated aerogels (**b**) Mechanism of fertilizer release from the aerogel in soil and water. Reprinted with permission from Ref. [115]. 2023, Elsevier.

Cellulose is the main component of plant cell walls, which has been extensively studied for delivery applications, including fertilizer delivery. Cellulose aerogel can be utilized as a carrier or matrix for controlled-release fertilizers, to release nutrients gradually over an extended period while ensuring optimal nutrient availability to plants while minimizing nutrient losses and environmental impact [110]. Owing to its ability to absorb and retain a significant amount of liquid fertilizers by impregnating the aerogel with liquid fertilizers, the nutrients can be stored within the aerogel matrix until the aerogel comes into contact with the soil or moisture. In a recent study, Zhang et al. [111] fabricated a simple approach for preparing amphoteric cellulose adsorbent by etherification of cellulose biopolymer with glycidylsulfonoic acid sodium salt and glycidyl trimethyl ammonium chloride in a one-pot reaction system. The authors achieved great adsorption capacity for sulfur and nitrogen (40.38 mg/g for NH_4_^+^ and 30.42 mg/g for H_2_PO_4_^−^.) into their system. The prepared aerogel was also able to be recovered and recycled after adsorption as a slow-release fertilizer. The porous structure of cellulose aerogel allows for the controlled release of absorbed fertilizers [112]. As the surrounding soil becomes moist, the nutrients held by the aerogel will gradually diffuse out, providing a steady supply of nutrients to the plants over time. Cellulose aerogel has a large surface area, which can promote interactions between fertilizers and plant roots [113]. This can enhance nutrient uptake efficiency by extending the contact time between the nutrients and the root system. Cellulose aerogel also exhibits excellent water-retention properties, which can help to maintain soil moisture levels and reduce water requirements [114]. This can be particularly beneficial in arid or water-limited regions, as it improves water-use efficiency and plant growth. In another study, Kaur et al. [115] developed a novel eco-friendly aerogel using waste hemp-stalk-derived nanocellulose for sustained and controlled fertilizer release. The authors converted cellulose to carboxymethyl cellulose, which was then cross-linked with green moiety and citric acid to produce a 3D aerogel system (Figure 5). Owing to its highly porous structure, large free volume amid polymeric chains, presence of hydrophilic groups, and high flexibility, the aerogel demonstrated super-absorbent behavior at neutral pH, absorbing 80 g/g of water within 27 h. Additionally, it exhibited sustained release properties for the encapsulated nutrients, urea, and ammonium dihydrogen phosphate. The authors also stated that the release kinetics of both fertilizers followed the Higuchi model and Fickian diffusion, confirming controlled-release mechanisms governed by dissolution and diffusion. Cellulose aerogel is derived from renewable sources and is biodegradable. Its use in fertilizer delivery systems aligns with sustainable agricultural practices, reducing the risk of pollution from excess fertilizer runoff [88]. It is important to note that while cellulose aerogel shows promise for fertilizer delivery, further research and development are required to optimize its performance and cost-effectiveness in practical applications [88,116]. Additionally, factors such as the specific fertilizer formulation, aerogel characteristics, and environmental conditions need to be considered when designing a cellulose aerogel-based fertilizer delivery system.

### 4.2. Alginate-Based Aerogel in Soil-Fertilizer Delivery

**Figure 6:**
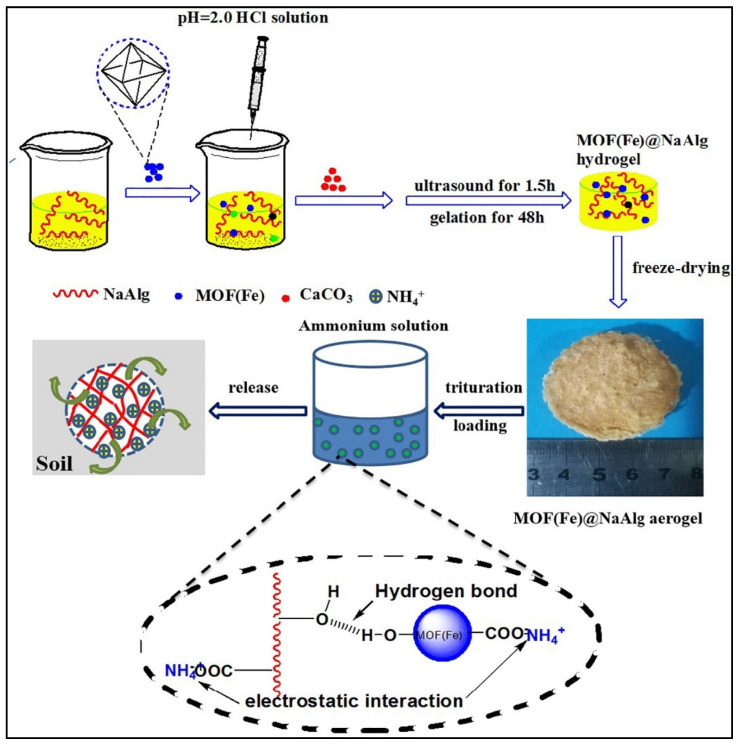
Illustration of the fabrication, fertilizer adsorption, and release from sodium alginate/metal–organic framework materials aerogel. Reprinted with permission from Ref. [123]. 2020, Elsevier.

Alginate is another biopolymer found in seaweed that has also been employed for soil-fertilizer delivery. Alginate aerogels possess several desirable characteristics that make them suitable for this purpose, including their highly porous structure with interconnected voids [117]. This structure enables the aerogel to absorb and retain a significant amount of liquid, including fertilizers and water, within its matrix [118]. The porous nature of alginate aerogels allows for the controlled release of fertilizers. Nutrients held within the aerogel matrix can gradually diffuse out when the aerogel comes into contact with moisture in the soil, ensuring a sustained and gradual supply of nutrients to the plants. Alginate aerogels exhibit excellent water-holding capacity, helping to retain moisture in the soil [119]. This can be particularly advantageous in arid or water-limited regions, as it enhances water-use efficiency and promotes plant growth. Liquid fertilizers can be mixed with the alginate solution before gelation, allowing the aerogel to absorb and encapsulate the nutrients. This ensures that the fertilizers are held within the aerogel matrix. In a recent study, Sun et al. [120] developed an alginate-based aerogel for the slow release of microencapsulated fertilizers. The prepared aerogel had a macroporous structure, in which nitrogen and phosphorus (the release elements) were scattered. The authors were able to obtain slow-release behavior after coating the fertilizers rather than the conventional adsorption process. It is well known that alginate aerogel, loaded with the desired fertilizer/s, can be added to the soil. As the soil becomes moist, the aerogel gradually releases the nutrients, providing a sustained nutrient supply to the plants. The release rate of nutrients can be influenced by various factors, such as aerogel composition, soil moisture, and environmental conditions [121,122]. Monitoring the nutrient levels in the soil and adjusting the dosage of the aerogel accordingly can help maintain an optimal nutrient balance for plant growth. Wu et al. [123] developed a novel sodium alginate-based aerogel using an ion cross-linking approach of metal–organic framework materials (MOFs) and sodium alginate (NaAlg) (Figure 6). The authors loaded ammonium (NH_4_^+^) as the desired fertilizer into their aerogel, which demonstrated an adsorption capacity of 29.4 mg/g and a swelling capability of 73 g/g. The same authors stated that the swelling behavior of the aerogel played a crucial role in the effective slow-release properties and the fertilizer formulation exhibited remarkable water-retention capacity in the soil. It is worth noting that the specific formulation and application of alginate aerogel for soil-fertilizer delivery may require further research and optimization to maximize its effectiveness and efficiency.

### 4.3. Other Biopolymer Aerogels in Soil-Fertilizer Delivery

Several studies have evaluated the use of other biopolymers in fertilizer delivery. Cheaper biopolymers such as chitosan and starch are commonly used to avoid the extra charges of the precursor materials. Chitosan is a biopolymer derived from chitin, which has been also employed in fertilizer delivery to enhance nutrient availability and control nutrient release. Chitosan aerogel exhibits a high absorption capacity, allowing it to absorb and retain liquid fertilizers within its porous structure. This property enables efficient nutrient loading and storage [124,125]. The porous nature of chitosan aerogel enables the controlled release of fertilizers. The absorbed nutrients can gradually diffuse out of the aerogel matrix when exposed to moisture in the soil. This controlled-release mechanism ensures a sustained and regulated supply of nutrients to the plants, reducing nutrient losses and optimizing nutrient uptake [33,112]. Chitosan can be also used to coat or encapsulate fertilizer granules or pellets. This coating acts as a protective barrier, preventing the rapid release of nutrients upon application [126]. It helps to control the release rate of nutrients and minimize nutrient leaching. Chitosan aerogel possesses excellent water-retention properties, helping to maintain soil moisture levels. This feature is beneficial for plant growth, especially in arid or water-limited regions, as it improves water-use efficiency and ensures adequate hydration for plants. In a recent investigation, Mutlaq et al. [127] used polysaccharide chitosan with poly (vinyl alcohol) and carboxymethyl cellulose to investigate its potential in fertilizer delivery. The authors reported that the prepared systems were able to absorb and retain large quantities of water, multiple times their weight, and were exploited for water retention when mixed with agricultural soil. In the soil–aerogel samples, the soil remained wet for nearly 40 days, whereas the reference samples (without aerogel) retained moisture for a maximum of 20 days only. Both aerogels exhibited high maximum loading percentages of NPK fertilizer and efficient loading percentages. The same authors reported that the release patterns of the loaded fertilizers from both aerogels demonstrated a controlled and sustained delivery of agrochemicals. When utilizing chitosan aerogel in fertilizer delivery and release systems, it is important to consider factors such as the formulation and composition of the aerogel, the specific fertilizers being used, soil conditions, and crop requirements [128]. Optimization and further research are ongoing to explore the full potential of chitosan aerogel in sustainable agriculture practices. It is important to note that the specific application and formulation of chitosan-based fertilizer delivery systems may require optimization based on factors such as the specific crop, soil conditions, and nutrient requirements. Additionally, further research and development are ongoing to explore the full potential of chitosan in fertilizer delivery and release for sustainable agriculture practices. Starch aerogel is another biopolymer aerogel that can be utilized for fertilizer-release applications.

**Figure 7:**
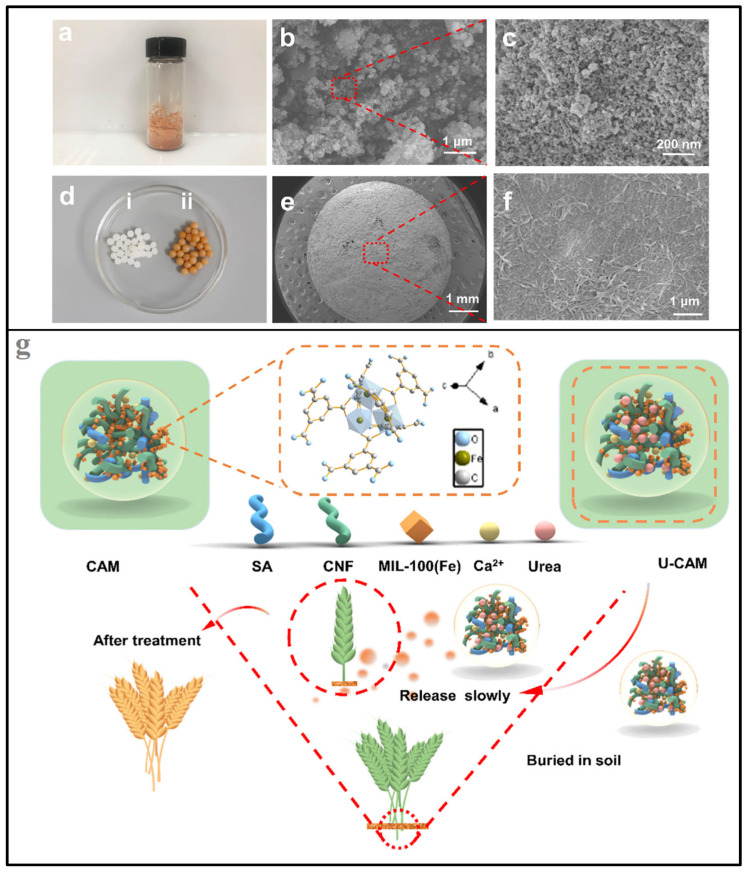
Illustration of a pH-responsive nanocellulose/sodium alginate/metal–organic frameworks aerogel and its application in the regulation of water and N-fertilizer; (**a**–**f**) Photograph and SEM images at different magnifications, and (**g**) simplified scheme of the fabrication approach and its application in urea-fertilizer slow release. Reprinted with permission from Ref. [133]. 2021, Elsevier.

Starch, a biopolymer derived from plant sources, can be processed into aerogel form to create a porous and lightweight material with desirable properties for fertilizer delivery [129]. The same as cellulose, starch can be used to encapsulate liquid fertilizers or nutrient solutions. The porous structure of the aerogel allows for the absorption and retention of the fertilizer within its matrix. Starch aerogels can provide a controlled-release mechanism for fertilizers [35]. The absorbed nutrients gradually diffuse out of the aerogel when exposed to moisture in the soil, ensuring a sustained and gradual release of nutrients to the plants [130]. The encapsulation of fertilizers within starch aerogel can help reduce leaching and nutrient loss. The aerogel acts as a protective barrier, preventing the rapid release of nutrients upon application and allowing for more efficient nutrient uptake by plants [131]. In one study, starch aerogel was evaluated for the slow release of fertilizers [132]. The authors used their aerogel to encapsulate urea (the fertilizer) into biomaterials using borax as a binder. The aerogel had a water retention ratio at 4 days of 14.76% in soil compared with the control which showed only 4.67% for soil. According to the study, the authors found that the standard fertilizer (without encapsulation) released approximately 97% of its nutrients within an hour when placed in distilled water. In contrast, the encapsulated fertilizers demonstrated slower release rates, with cumulative releases of 76% and 68% at 24 h and 96 h. Notably, when applied to the soil, aerogel exhibited sustained release patterns, with cumulative urea releases of approximately 95% and 92% after 36 days. Starch aerogels have the ability to retain water, contributing to improved moisture levels in the soil. This can be particularly beneficial in dry or arid environments, where it helps enhance water-use efficiency and supports plant growth. The formulation and application of starch aerogels for fertilizer release may require optimization based on specific fertilizer types, soil conditions, and crop requirements. Further research and development are ongoing to explore the full potential of starch aerogels in fertilizer delivery systems, aiming to enhance nutrient availability and promote sustainable agriculture practices. Wang et al. [133] developed a pH-sensitive nanocellulose/MOFs aerogel by combining the nanocellulose aerogel with MIL-100 (Fe), resulting in an aerogel with a substantially high surface area (Figure 7). The authors stated that the highest water adsorption (100 g/g) was achieved at pH 11, highlighting the aerogel’s ability to absorb and retain water effectively. The release of urea at pH 11 occurred at a much slower rate compared to that at pH 3, indicating its potential suitability for application in arid regions. These results confirmed the beneficial effects of such aerogels on crop growth, validating their potential for further application in irrigation-based farming. These findings also open up possibilities for their application in addressing water eutrophication concerns and improving agricultural practices.

## 5. Future Directions and Challenges of Biopolymer Aerogels in Soil-Fertilizer Delivery

The future directions of biopolymer aerogels in soil-fertilizer delivery are centered around improving their performance and scalability and understanding their environmental impact. Further research is needed to fine-tune the composition and structure of biopolymer aerogels to achieve precise and customizable nutrient-release profiles. Understanding the mechanisms of nutrient diffusion within the aerogel matrix and optimizing the interactions between the aerogel and the fertilizer will be crucial in developing aerogels that can release nutrients according to specific crop requirements. Researchers are working on modifying the properties of biopolymer aerogels to enhance their nutrient-loading capacity, mechanical strength, and water-absorption capabilities. By adjusting parameters, such as cross-linking density, porosity, and surface chemistry, aerogels can be optimized for specific soil and crop types. The use of sensors and smart monitoring systems can be combined with biopolymer aerogels to provide real-time data on soil moisture, nutrient levels, and plant health. This integration would enable precise and targeted nutrient delivery based on the plant’s needs, leading to improved efficiency and reduced waste. One of the challenges in the widespread adoption of biopolymer aerogels is the scalability of their production. Research efforts are focused on developing cost-effective and large-scale manufacturing processes for biopolymer aerogels, ensuring their commercial viability for widespread agricultural use. While biopolymer aerogels are generally considered environmentally friendly, it is important to conduct comprehensive assessments of their long-term impact on soil health, microbial communities, and overall ecosystem dynamics. This includes investigating their biodegradability, potential leaching of chemicals, and any unintended consequences on soil structure and biodiversity. Further field trials and long-term studies are needed to evaluate the performance of biopolymer aerogels in different soil and climate conditions, as well as various crop types. These trials will provide valuable insights into the effectiveness, economic viability, and environmental sustainability of biopolymer aerogels as soil-fertilizer delivery systems. As biopolymer aerogels gain traction in agriculture, regulatory frameworks will need to be developed to ensure their safe and responsible use. This may involve establishing guidelines for product labeling, application rates, and disposal practices to minimize any potential risks to human health and the environment. Addressing these future directions and challenges will contribute to the continued advancement and successful implementation of biopolymer aerogels in soil-fertilizer delivery, ultimately promoting sustainable agriculture practices and minimizing the environmental impact of conventional fertilizer application. It is worth noting that while biopolymer aerogels show great potential, further research and development are still needed to optimize their properties and understand their long-term effects on soil health and plant growth. However, their unique characteristics make them an exciting area of study for sustainable agriculture and fertilizer delivery systems.

## Figures and Tables

**Table 1:** Illustration of the properties of different biopolymer aerogels.

Precursor Material	Cross-Linker	Drying Technique	Density (mg/cm^3^)	Porosity (%)	Surface Area (m^2^/g)	Application	Ref
Cellulose nanofibers	Pure CNF	Freeze-drying	8.1 ± 0.8	99.1 ± 0.2	145.3 ± 2.7	Biomedical	[58]
Chitosan	Pure chitosan	Freeze-drying	141.4	90.8	-	General applications	[59]
Sodium alginates	Calcium chloride	Supercritical drying	-	98	402 ± 12	Drug delivery	[60]
Gelatin	Glutaraldehyde	Freeze-drying	115	75.37	9.56	Oil–water separation	[61]
Starch	Corn starch	Supercritical fluid	100.6	90.1	240	Drug delivery of active	[62]
Cellulose nanocrystals	Poly (vinyl alcohol)	Freeze-drying	22.5	97.7	38.0	water–oil separation	[63]
Starch	Pure starch	Supercritical drying	602	-	90.0	Vitamins delivery	[64]
Carrageenan	Potassium chloride	Supercritical CO_2_	250.0	86.0	110.0	Drug delivery	[65]
Biomass	Phytic acid	Freeze-drying	52.0	-	-	Thermal insulation	[42]

## Data Availability

All the data is available within the text.
